# Characteristics of and risk factors for severe neurological deficit in patients with pyogenic vertebral osteomyelitis: A case–control study

**DOI:** 10.1097/MD.0000000000006387

**Published:** 2017-05-26

**Authors:** Adrien Lemaignen, Idir Ghout, Aurélien Dinh, Guillaume Gras, Bruno Fantin, Virginie Zarrouk, Robert Carlier, Jean-Edouard Loret, Eric Denes, Alix Greder, François-Xavier Lescure, David Boutoille, Pierre Tattevin, Bertrand Issartel, Jean-Philippe Cottier, Louis Bernard

**Affiliations:** a Department of Infectious Diseases, University Hospital of Tours, Francois Rabelais University, Tours; b Clinical Research Unit, University Hospital A. Paré, APHP, Boulogne; c Infectious Diseases Unit, University Hospital R. Poincaré, APHP, Versailles Saint Quentin University, Garches; d Department of Internal Medicine, University Hospital Beaujon, APHP, Clichy; e Radiology Department, Neuro-musculoskeletal Pole, University Hospital R Poincaré, APHP, Versailles University, Paris-Saclay UMR 1179, Garches; f Department of Neurosurgery University Hospital of Tours, Tours; g Department of Infectious Diseases, University Hospital of Limoges, Limoges; h Department of Infectious Diseases, Mignot Hospital, Versailles; i Department of Infectious Diseases, Bichat-Claude Bernard University Hospital, APHP, Paris; j Department of Infectious Diseases, Hotel-Dieu University Hospital, Nantes; k Infectious Diseases and Intensive Care Unit, Pontchaillou University Hospital, Rennes; l Department of Infectious Diseases, Clinique du Parc, Lyon; m Department of Neuroradiology, University Hospital of Tours, Francois Rabelais University, Tours, France.

**Keywords:** neurologic manifestations, risk factors, spinal cord compression, vertebral osteomyelitis

## Abstract

Severe neurological deficit (SND) is a rare but major complication of pyogenic vertebral osteomyelitis (PVO). We aimed to determine the risk factors and the variables associated with clinical improvement for SND during PVO.

This case–control study included patients without PVO-associated SND enrolled in a prospective randomized antibiotic duration study, and patients with PVO-associated SND managed in 8 French referral centers. Risk factors for SND were determined by logistic regression.

Ninety-seven patients with PVO-associated SND cases, and 297 controls were included. Risk factors for SND were epidural abscess [adjusted odds ratio, aOR 8.9 (3.8–21)], cervical [aOR 8.2 (2.8–24)], and/or thoracic involvement [aOR 14.8 (5.6–39)], *Staphylococcus aureus* PVO [aOR 2.5 (1.1–5.3)], and C-reactive protein (CRP) >150 mg/L [aOR 4.1 (1.9–9)]. Among the 81 patients with PVO-associated SND who were evaluated at 3 months, 62% had a favorable outcome, defined as a modified Rankin score ≤ 3. No factor was found significantly associated with good outcome, whereas high Charlson index [adjusted Hazard Ratio (aHR) 0.3 (0.1–0.9)], low American Spinal Injury Association (ASIA) impairment scale at diagnosis [aHR 0.4 (0.2–0.9)], and thoracic spinal cord compression [aHR 0.2 (0.08–0.5)] were associated with poor outcome. Duration of antibiotic treatment was not associated with functional outcome.

SND is more common in cervical, thoracic, and *S. aureus* PVO, in the presence of epidural abscess, and when CRP >150 mg/L. Although neurological deterioration occurs in 30% of patients in early follow-up, the functional outcome is quite favorable in most cases after 3 months. The precise impact of optimal surgery and/or corticosteroids therapy must be specified by further studies.

## Abstract 2

Supplemental Digital Content is available in the text

## 1 Introduction

Pyogenic vertebral osteomyelitis (PVO) is the leading cause of hematogenous osteomyelitis in adults over 50 years old.^[1]^ The estimated incidence of PVO is between 0.5 and 10 per 100 000 inhabitants per year in Europe.^[1–4]^ Incidence has been increasing over the last 15 years probably due to higher frequency of aging and comorbid patients in the general population and improvement of diagnostic techniques.^[3,4]^

The incidence of neurological complications in PVO varies and depends mainly on the criteria used to define a neurological complication. Up to 30% of patients present any neurological complication, encompassing radicular pain, sensitive disorder, sphincter abnormalities, or motor weakness.^[5–8]^ The latter occurs in 5% to 25% of patients with PVO.^[9–12]^ Neurological deficits persist in about 30% of cases presenting with any neurological complication at diagnosis, and in about 60% of cases presenting with motor weakness.^[7,9,13]^ The factors associated with severe neurological deficit (SND) in PVO remain unclear. Early identification of patients at risk of SND could contribute to optimize patient monitoring and management.

Here, we describe clinical, microbiological, biological, and radiological factors associated with SND in PVO and identify factors associated with outcome in patients with PVO-associated SND.

## 2 Methods

### 2.1 Definitions

PVO cases in adult patients were defined as the presence of osteomyelitis affecting the disc, vertebral body, or both, at one or multiple sites, in association with significant microbiological documentation by positive blood culture, radiologically guided disco-vertebral biopsy or samples obtained during vertebral surgery.

SND was defined as the appearance of new motor weakness at any time during follow-up from the first symptoms of PVO. Motor weakness had to be recorded by a physician, with a motor score inferior to 4/5^[14]^ for at least 1 muscular group.

### 2.2 Study design and data collection

We conducted a case–control study to identify variables associated with SND in PVO. Patient data were in part extracted from the previously published randomized controlled trial NCT00764114 (RCT) investigating optimal duration of treatment in PVO.^[8]^ Briefly, 351 adults with definite diagnosis of PVO from 71 medical care centers in France were randomly assigned to either 6 or 12 weeks of antibiotic treatment, between 2006 and 2011. From these patients, those presenting with SND were included as cases and those without SND were included as controls in the present study (trial group). To increase the power of the study, we retrospectively collected supplementary data from patients presenting PVO with SND from 8 medical care centers in France between 2001 and 2013 following the same criteria for case definition (retrospective group). To screen patients, we used an algorithm searching for the combination of “pyogenic vertebral osteomyelitis” and “neurological symptoms” in the French Hospital Discharge Database of each center.^[2]^

Screened files were reviewed to select eligible patients. Apart from presence of SND, inclusion and exclusion criteria were identical in the trial group and the retrospective group. Patient information was collected from medical records through a standardized data collection form. Patients presenting a postoperative PVO were excluded.

### 2.3 Objectives

The main objective of this study was to describe clinical, microbiological, biological, and radiological factors associated with SND in PVO.

We conducted a secondary descriptive analysis on the subgroup of patients presenting a PVO with SND. Objectives were to describe characteristics of neurological complications and their management and to identify factors associated with a good functional outcome over time for patients with motor weakness.

### 2.4 Variables of interest

Background variables included age, gender, modified Charlson index,^[15]^ and comorbidities (diabetes mellitus, smoking history, or immunodeficiency). Variables related to clinical, biological, and radiological presentation at the time of diagnosis of PVO were collected: time to diagnosis from the first symptom (defined by the date of occurrence of pain, fever and/or chills), fever or pain at diagnosis, initial C-reactive protein (CRP) peak, type and results of imaging (site affected, spinal cord ischemia, presence of epidural abscess, as defined by Ledermann et al),^[16]^ and detailed neurological examination. For patients with worsening of neurological symptoms during follow-up, time from diagnosis to worsening was recorded. Motor weakness was assessed using the American Spinal Injury Association impairment scale (AIS), taking into account the lowest score during follow-up.^[17]^ Worst score is A, corresponding to a complete spinal cord injury.

For patients with SND, we considered the following treatment and follow-up variables: whether surgery was performed or not, type of surgery (laminectomy, arthrectomy, epidural drainage, and arthrodesis), duration of antibiotic treatment, outcome at last follow-up, with AIS assessment, and modified Rankin score.

Favorable functional outcome at the last follow-up was defined as a modified Rankin score of 3 (favorable) or less (unfavorable), indicating the ability to walk without assistance.^[18]^

### 2.5 Number of subjects

We considered the 351 patients included in the RCT as potential controls. Previous studies found that neurological dysfunction is associated with involvement of the thoracic spine.^[5,9]^ Given that involvement of the thoracic spine was expected in about 30% of controls^[6]^ and 60% of cases,^[9]^ 1 case for 3 controls was necessary to find a significant difference with an odds ratio (OR) of 1.9 with 80% power and an α risk of 5%.

### 2.6 Statistical analyses

All statistical analyses were performed using R software version 3.1 (R: A language and environment for statistical computing; R Foundation for Statistical Computing, Vienna, Austria). Data were described by group using the appropriate statistical parameters. Distributions were compared using Chi-square test or Fisher exact test if necessary. Continuous quantitative variables and ordinal variables were tested using the nonparametric method of Kruskal–Wallis. To identify risk factors for severe neurological signs, a multivariable logistic regression model was developed. We checked if any independent variable in the model was determined by a linear combination of other independent variables. The generalized variance-inflation factors calculated for *Staphylococcus aureus*, epidural abscess, positive blood cultures, and CRP over 150 mg/L (1.04, 1.1, 1.16, and 1.15, respectively) were low, indicating the absence of multicollinearity. Explanatory variables included in the model were those giving a degree of significance (*P*) below 5% in univariate analysis. The final model was developed by a selection of step-down approaches using Akaike criterion. Missing data were imputed by a chained equation using the MICE package in R (Multivariate Imputation by Chained Equations in R, S. van Buuren and K. Groothuis-Oudshoorn, J Stat Soft, 2011;45:1–67) in a sensitivity analysis, using the final model to ascertain the robustness of estimates.

For secondary analyses, we excluded from the subgroup of patients presenting PVO with SND without neurological evolution follow-up. To deal with multiple vertebral level affected cases in multivariable analysis, we considered only the level of the clinical spinal cord compression. A Cox proportional hazards model was performed to identify factors associated with functional outcome. We defined the critical event as the first date on which was recorded a Rankin score of 3 or less. Variables were included in the model if they had a degree of significance below 5% in the univariate analysis apart for duration of antibiotic treatment which was forced. The final model was selected with the best goodness of fit.

## 3 Results

### 3.1 Population description

**Figure 1:**
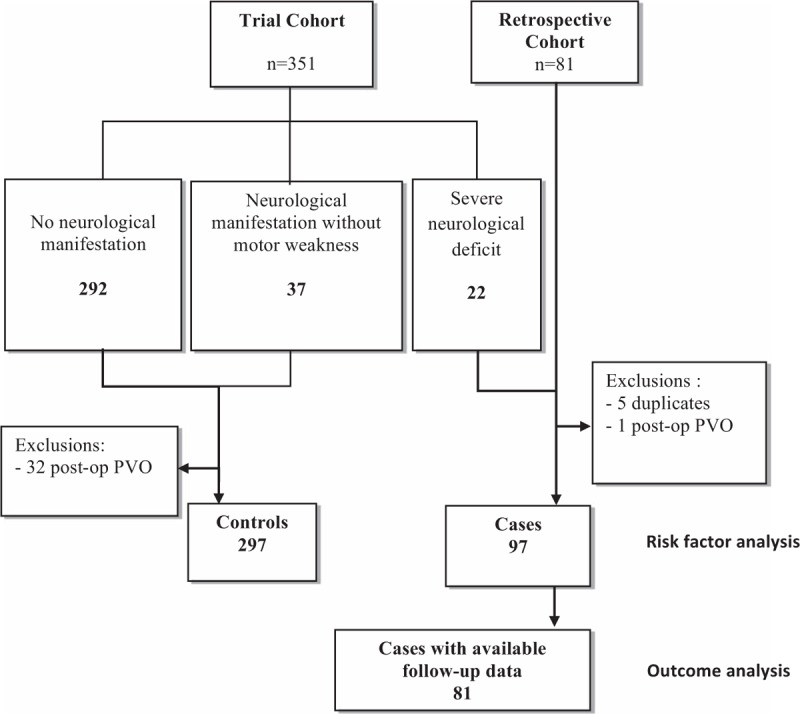
Flow-chart of 394 patients with pyogenic vertebral osteomyelitis with (cases) or without (controls) severe neurological deficit. Trial Cohort: Patients from randomized trial NCT00764114, Retrospective cohort: Cases included from the French Hospital Discharge Database, Risk factor analysis: patients included for descriptive analysis of evolution and treatment, Outcome analysis: patients included in the analysis of neurological outcome at 3 months. Post-op PVO = postoperative pyogenic vertebral osteomyelitis.

Of 351 patients included from the randomized trial, 318 met the inclusion criteria; 21 patients presented SND and were classified as cases and the other 297 were included as controls. These 21 patients were pooled to 81 cases with PVO and SND identified from the French Hospital Discharge Database (retrospective group; Fig. 1) constituting a group of 97 cases with SND. Concerning the 97 patients with SND, 81 had clinical data available for a descriptive analysis (particularly neurological follow >3 months). There was no significant difference between cases from the 2 pooled patient groups with PVO and SND (see Supplementary 1). In the global population of our study, the median time (±SD) between first symptoms (back pain, fever, or chills) and confirmation of diagnosis of PVO was 39.6 days (±65.4). Clinical, microbiological, and radiological characteristics of all included patients are summarized in Table 1.

**Table 1:**
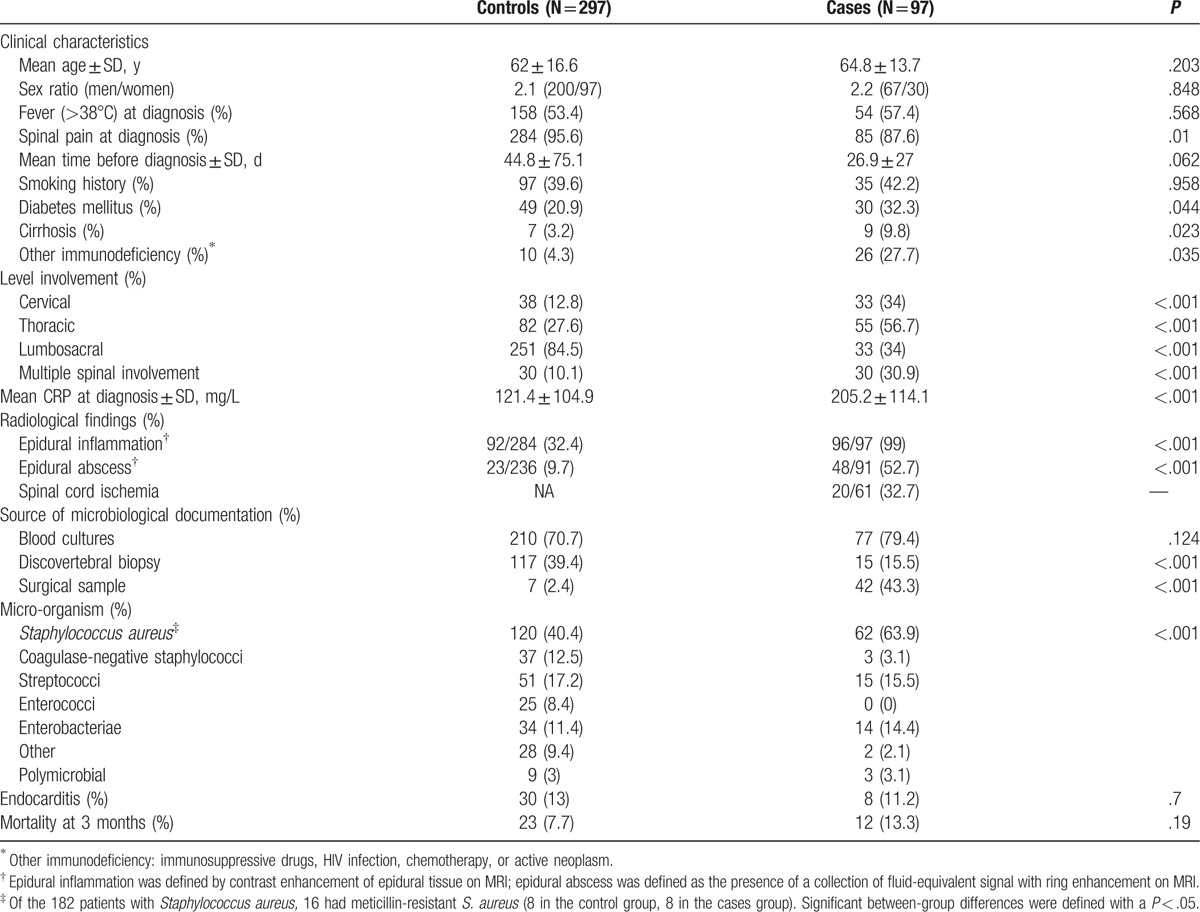
Characteristics of patients with pyogenic vertebral osteomyelitis, with (cases) or without (controls) severe neurological impairment.

### 3.2 Comparison of characteristics between PVO patients with and without SND

The 2 groups were similar with regard to age, sex ratio, median time between first symptoms and confirmation of diagnosis, presence of fever at diagnosis, positive blood culture (Table 1). The median CRP peak level, the involvement of multiple spinal sites, the thoracic and cervical localization, presence of epidural abscess, *S. aureus* were more significantly associated with cases of SND than controls, whereas spinal pain was more frequent in controls (88% vs 96% for cases and controls, *P* = .01).

### 3.3 Variables associated with SND

Risk factors for SND were epidural abscess [adjusted odds ratio, aOR 8.9 (3.8–21)], cervical [aOR 8.2 (2.8–24)], and/or thoracic involvement [aOR 14.8 (5.6–39)], *S. aureus* PVO [aOR 2.5 (1.1–5.3)], and CRP >150 mg/L [aOR 4.1 (1.9–9)] (Table 2). Results of sensitivity analysis on the whole population of 97 cases versus 297 controls using multiple imputation of missing data were consistent with the main analysis (see Supplementary 2).

**Table 2:**
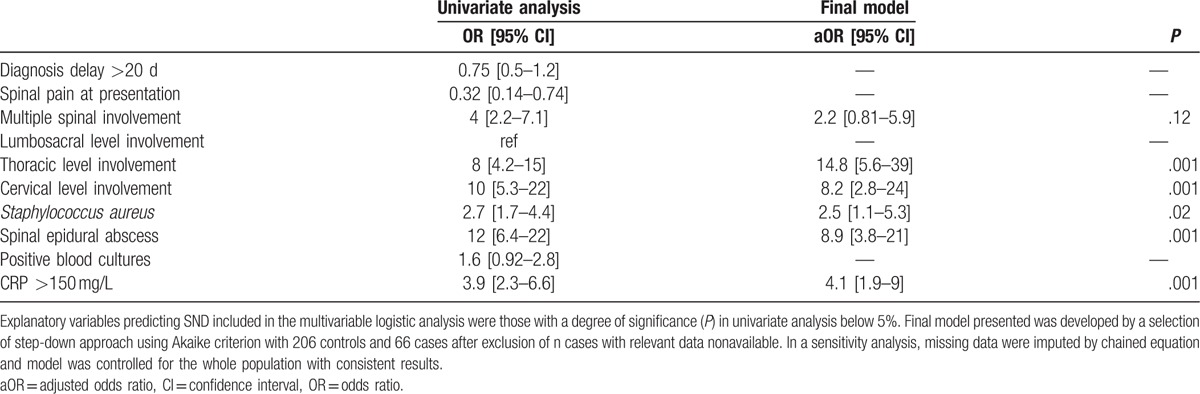
Factors associated with severe neurological deficit in patients with pyogenic vertebral osteomyelitis.

### 3.4 Detailed description of 81 patients with SND

Neurological signs were present in 71 of 81 (87.6%) of patients at admission, of whom 65 of 71 (80%) had motor weakness. The main sites affected were the thoracic spine (35/65, 53.8%). Six patients had first an isolated neurological symptom: radicular pain or sphincter dysfunction. Median time from first general symptoms (back pain, fever, or chills) to diagnosis of PVO was shorter for the 71 patients presenting with neurological signs at diagnosis [18 days; interquartile range (IQR), 9–24 days], and 59.5 days (IQR, 39–77 days) otherwise (n = 10), *P* = .005.

During follow-up in hospitalization, 26 cases (32%) experienced a worsening of pre-existing neurologic signs (16 patients) or the appearance of a new motor weakness (10 patients). For these 26 patients, median time to onset of new signs since the diagnosis of PVO was 11 days (IQR, 4–24 days).

The causes of worsening were unfavorable local evolution under treatment for 19 of 26 (76%); mechanical compression after a brutal backward shift of weakened posterior spine wall into the medullary canal following a fall in 2 patients; and development of a motor deficit after surgery for 4 patients (2 related to postoperative spinal hematoma and 2 related to a post-laminectomy spinal dislocation). Overall, the site of medullary compression was lumbosacral in 19 patients (23.5%), including 1 with an AIS of A or B, thoracic in 41 patients (50, 6%) including 23 with AIS of A or B and cervical in 25 (25.9%) including 11 with AIS of A or B.

### 3.5 Therapeutic management of patients with SND

Of the 81 patients, 38.3% had received antibiotics before diagnosis of PVO (31/81). The median duration of antibiotic treatment was 90 days (IQR, 45–90 days), and 22 patients (27.1%) received less than 45 days of antibiotic treatment. Antibiotic treatment varied according to microbiology (*S. aureus*: intravenous anti-staphylococcal betalactam, switch to a combination of fluoroquinolone and rifampicin; *Enterobacteriae:* third-generation cephalosporins and/or fluoroquinolone; Streptococcal: amoxicillin). Seventy-three patients (91%) were temporarily immobilized, with a corset when technically feasible.

Surgery was performed in 58 of 81 (71.6%) patients. The main indication was neurological impairment. Median time to surgery from the medical diagnosis of motor weakness was 2 days (IQR, 1–3 days). The most common type of surgical procedure was spinal decompression by posterior approach without fixation (39/58, 67.2%), including 36 laminectomy. An anterior approach with fixation was used in 8 patients, for whom the cervical spine or cervicothoracic hinge was affected. A posterior approach with fixation was used in 11 patients, mostly in cases with thoracic spine involvement.

Patients with more severe motor impairment were less likely to receive decompressive surgery: 40% of patients with an AIS of A, 81% if AIS was B, 65% with AIS of C, and 82% with AIS of D (*P* = .055).

### 3.6 Outcome of patients with SND

Median follow-up was 135 days (IQR, 82–233). Of the 81 patients with PVO-associated SND, 9 (11.1%) died during the first 3 months, including 6 directly attributable to PVO-related sepsis, or as a complication of SND. One documented microbiological failure occurred in a 78-year-old man with lumbar PVO due to meticillin-resistant *S. aureus*, who relapsed after a 6-month course of appropriate antibacterial treatment, in the absence of surgery.

An improvement in neurologic examination defined by improvement of AIS at 3 months was observed in 45 of 73 (62%) of cases.

**Figure 2:**
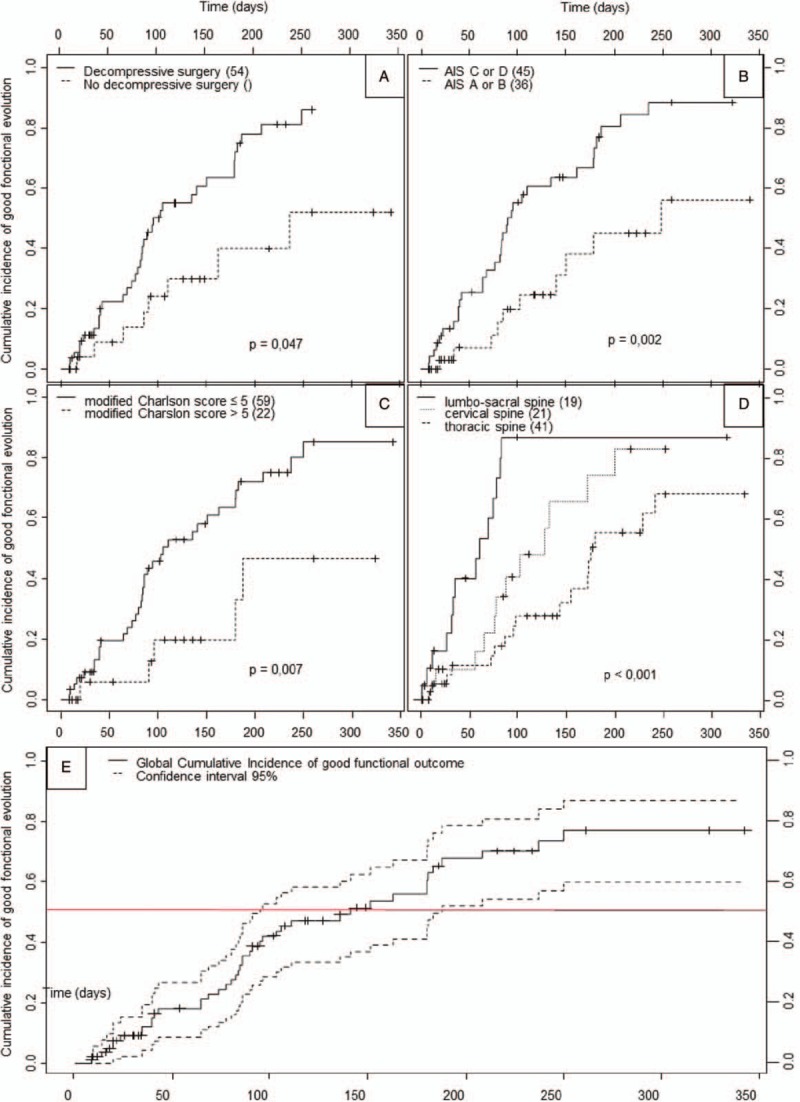
Functional outcome according to parameters significant in univariate analysis, and representation of global model. Good functional evolution was defined by a Rankin score of 3 or better indicating the ability of walking without human help. Each square represents cumulative incidence of good functional evolution over time according to each studied variable, with associated global *P* value in univariate analysis. In brackets are the effectives of each subgroup. (A) Outcome whether decompressive surgery is performed or not (isolated arthrodesis was not considered as decompressive); (B) Outcome according to the worst AIS at installation of neurological impairment; (C) Outcome according to modified Charlson score with a cut-off at 5; (D) Outcome according to spine level of compression; (E) Graphical representation of final Cox proportional hazards model with confidence interval and 50% cumulative incidence line.

Median Rankin score at 3 months was 3 (IQR, 2–5), and at the end of follow-up, a favorable functional outcome was reached by nearly 80% of patients. The median cumulative incidence of Rankin score of 3 or less occurred after 140 days (IQR, 77–249) (Fig. 2). With a median follow-up of 130 days, none of the 10 patients with an initial AIS of A were able to walk without help and only 2 had a better AIS. Factors significantly associated with functional outcome in univariate analysis are depicted in Fig. 2. The presence of spinal cord ischemia was significantly associated with a poor outcome, but insufficiently recorded to be included in multivariable analysis [13/20 (62%) had a Rankin score >3 at last follow-up, vs 12/41 (29%) when spinal cord ischemia was absent, *P* = .008]. Microbiology sample and presence of an epidural abscess were not associated with functional outcome. Neither were diabetes mellitus and older age on their own, but these variables were included in the modified Charlson score. It is noteworthy that duration of antibiotic treatment was not associated with functional outcome, and there was no significant difference between patients treated less or more than 6 weeks (22 and 56 patients, respectively, 3 patients with unavailable data).

The final Cox proportional hazards model found 3 factors significantly associated with poor functional outcome over time: an adjusted Charlson index above 5 [hazard ratio: HR = 0.30 (0.10–0.88)], the severity of initial motor weakness [AIS A or B vs C or D: HR = 0.43 (0.20–0.92)], and the level of spine compression [thoracic level: HR = 0.19 (0.08–0.46); cervical level: HR = 0.34 (0.14–0.84)]. Decompressive surgery appeared protective in univariate analysis, but did not reach significance in multivariate analysis [HR = 0.43 (0.18–1.05), *P* = .06].

## 4 Discussion

In this large, retrospective, multicenter study, our results showed that PVO complicated with SND are more common in thoracic PVO, with infection due to *S. aureus*, in the presence of epidural abscess, in the presence of CRP level >150 mg/L. Although neurological deterioration occurs in 30% of patients during PVO complicated with SND, the functional outcome is favorable in nearly 80% of patients, especially those who underwent decompressive surgery.

This study has several limitations. In order to be as homogeneous as possible, we excluded tuberculous, brucellar, fungal, and postoperative infections.^[19,20]^ We defined SND as the presence of motor weakness because this is the most consistent criterion among neurological manifestations.^[5,6,10,11]^ The choice of a case–control study to identify risk factors for motor weakness in PVO was dictated by the rarity of the disease. To improve the power of the study, we decided to add cases from a large prospective data collection to a retrospective cohort from the French database. Thereby, there was no significant difference between the 2 collections. Another limitation is the missing data during follow-up of patients issue from a retrospective cohort (French database). Missing data for the variables included in the final model did not exceed 18%. Multiple imputation by chained equation was used to reduce the risk of bias induced by missing values and showed the same results.

Patients with SND had neurological signs at admission in 87.6% of cases. An important observation in this study was the occurrence or worsening of neurological impairment in almost one-third of patients after PVO diagnosis, mostly before any surgical procedure, and in 12.4% of cases, motor impairment appeared during hospitalization. This worsening of neurological condition, described in another study,^[21]^ was observed in different situations, mostly in case of noncontrolled infection but also due to postoperative impairment or secondary mechanical complication. SND can occur during hospitalization, even late (up to 24 days), so we therefore recommend examining at-risk patients carefully, at least twice daily during the first days.

Several mechanisms responsible for the appearance of the SND during PVO may be isolated or associated: epidural abscess in the narrowing of the spinal space, septic embolization of the vertebral artery, spinal cord ischemia, and septic vertebral fracture with medullar mechanical compression.

**Figure 3:**
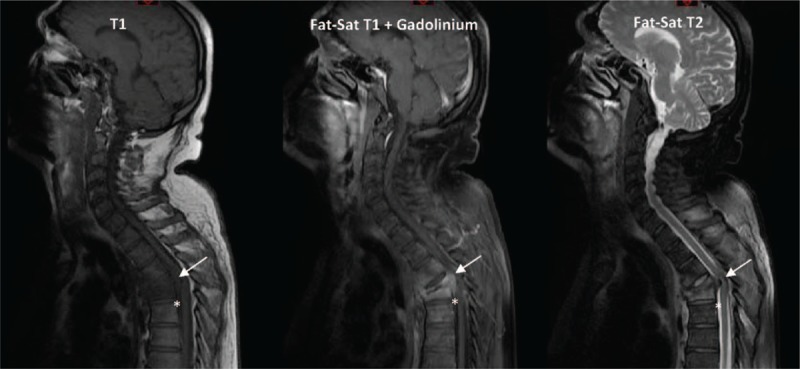
Magnetic resonance imaging (MRI) in pyogenic vertebral osteomyelitis. Fat-Sat: T1 or T2 sequence with saturation of fat signal. Vertebral osteomyelitis show intervertebral disc and adjacent vertebrae hyposignal on T1 sequence, contrast enhancement in T1 sequence with Gadolinium injection. Nucleus of intervertebral disc and adjacent vertebrae appear in hypersignal on T2 sequence. T4-T5 pyogenic vertebral osteomyelitis with disc disappearance, vertebral body destruction, kyphotic angulation, epidural inflammation (^∗^), and spinal cord compression by an anterior epidural abscess (arrow).

Almost all patients with motor weakness had epidural inflammation on magnetic resonance imaging (MRI; elsewhere called epidural phlegmon) whether associated or not with epidural abscess, whereas this radiological abnormality was present in only 92 of 297 controls (30.1%). Thus, epidural inflammation may be used to identify patients at a high risk of SND, who would require closer monitoring (Fig. 3).

Upper spinal site of infection was previously identified as a factor associated with paralysis.^[5,9]^ In our study, involvement of the thoracic spine was associated with the higher risk of SND. Thickness of the medullary canal and scarcity of artery supply at this site could explain this observation. Cauda equine syndrome was infrequent in respect to the number of patients with involvement of the lumbosacral spine.

Indeed, a huge epidural abscess or extensive epidural abscess is required to induce radicular compression due to the width of medullary canal at this location, and higher compressibility of nerve roots of the cauda equine.^[22]^

The presence of a central hyperintensivity of the spinal cord on MRI, reflecting medullary suffering, was associated with poor outcome in a univariate analysis. We did not include this variable in the multivariable analysis because this information was available in only 75% of cases. The mechanism of spinal cord suffering is unclear, but could be due to medullary ischemia, with or without para-infectious vasculitis.^[23]^

Finally, some precisions in MRI findings were not recorded and therefore could not be analyzed in our risk factor analysis. In the work from Bart et al,^[21]^ the presence of an epidural abscess was not associated with SND while cervical lesions, destruction grade 3 of the above vertebrae, destruction of posterior arch, dural sac stenosis, spinal cord hypersignal, anterior effacement of the subarachnoid space, and angular kyphosis deformity were significantly associated with SND. This difference is possibly due to insufficient precision in our data collection, but in our experience, the presence of an epidural abscess in PVO is often associated with dural sac stenosis, spinal cord hypersignal, or anterior effacement of the subarachnoid space.

In addition to these known factors, the high level of initial biological inflammatory response (CRP level >150 mg/L) and the involvement of *S. aureus* were associated with motor weakness. Diagnosis delay was not significantly associated with complications probably because of the more aggressive presentation associated with motor weakness, with virulent micro-organisms and “noisy” clinico-biological presentation. Of note, *S. aureus* was not associated significantly with poor functional outcome or difficult-to-treat PVO. Among patients presenting SND, infections due to *S. aureus* had the same characteristics, presentation and outcome as those due to other micro-organism, except for more diabetes and more frequent positive blood cultures with *S. aureus*. Unlike other studies, we did not find an association between SND and underlying conditions or immunosuppression.

Regarding the prognostic factors, functional outcome appeared more relevant due to the low number of deaths or microbiological failures. We used the modified Rankin scale to assess functional outcome, although it was designed for cerebral injuries. Its simplicity and reproducibility was adapted for a retrospective record, including for spinal cord compressions.^[24]^ A score of 3 or less was used as a good functional outcome because it ensures a relative independent mobility. Survival analysis was used in order to minimize loosening of information, as median follow-up of cases was only 4.5 months (IQR 2.7–7.8). Long-term outcome was studied elsewhere with conflicting results.^[13]^

Outcome in our patients was mainly associated with severity of initial neurological impairment and underlying conditions. The only modifiable prognostic factor was the surgical management, inline with other findings^[1,7,9]^ but was not confirmed in multivariate analysis. In our study, instrumental fixation seemed to be associated with good functional outcomes similar to the study by Rath et al,^[25]^ but 4 patients (7%) have developed a motor deficit after surgery. These techniques are used mainly for mechanical instability or neurological threat, and safety seems acceptable, including during the acute phase of PVO-related sepsis.^[26–28]^

An important finding was the absence of a clear benefit related to prolonged antibiotic treatment, neither on microbiological failure or functional outcome, even in the setting of severe PVO with paravertebral abscess. Antibacterial treatment must be optimized, particularly when Methicillin resistant Staphylococcus aureus is involved, or with high inoculum, but there is no scientific evidence to promote prolonged antibiotic therapy in patients with PVO-remoted SND.^[8,29]^

Two-third of patients had a neurological favorable outcome at 3 months. The physical medicine and rehabilitation has probably a prevailing role in the improvement of the functional prognosis in this disease,^[30]^ but we have not evaluated this point in our retrospective study.

Systemic corticosteroids could be a therapeutic option in these cases, as for tuberculous vertebral osteomyelitis.^[24]^ Steroids were used for only 10 patients in our series, hence we could not include this variable in the multivariable analysis. However, none of these 10 patients who received systemic corticosteroids for PVO-related SND died or had worsening of neurological impairment, and 6 of them (60%) were significantly improved.

## 5 Conclusion

SND in PVO occurs in 6% of cases and is associated with considerable morbidity. Factors associated with SND are involvement of upper spine (cervical or thoracic), high level of CRP at diagnosis, implication of *S. aureus*, and the presence of epidural abscess on MRI. Patients should be carefully checked during initial hospitalization, as up to 30% of patients with SND developed or worsened motor weakness during follow-up, with a median delay of 11 days from PVO diagnosis. Outcome is quite favorable, with most patients recovering autonomous walk after several months. Poor functional outcome, defined by the inability to walk without help, was associated with severity of initial motor weakness, high adjusted Charlson index, and spinal cord compression at thoracic level, whereas surgery seems to be protective.

## Acknowledgments

We thank all patients for participation in this study. We thank the staff (physician, nurses, medical microbiologists, secretaries, and assistants) in each participating center for their help and cooperation. We thank Audrey Barrelet and Grégoire Lambert de Cursay for their help in medical records.

We also thank the “Centre de Reference pour les infections Osteo-articulaires du Grand-Ouest de la France” (CRIOGO) and the “Groupe d’épidémiologie et de recherche et d’investigation Clinique du Centre Ouest” (GERICCO) for their contribution to this study.

## Supplementary Material

Supplemental Digital Content
