# Supplementary material for: Characteristics of and risk factors for severe neurological deficit in patients with pyogenic vertebral osteomyelitis: A case–control study
Source: Medicine (Baltimore). 2017 May 26;96(21):e6387. doi: 10.1097/MD.0000000000006387 (PMC5457841; doi:10.1097/MD.0000000000006387)
Supplement: Supplemental Digital Content [file medi-96-e6387-s001.docx]

**Supplementary Data**

**Supplement 1: Comparison of patients with pyogenic vertebral osteomyelitis and severe neurological deficit in the two subgroups**

**Supplement 2: Sensitivity analysis with multiple imputations**

**Supplement 1: Comparison of patients with pyogenic vertebral osteomyelitis (PVO) and severe neurological deficit (SND) in the two subgroups**

|  | **Trial** | **Retro** | **p** |  |
| --- | --- | --- | --- | --- |
|  | (N=21) | (N=76) |  |  |
| Clinical characteristics |  |  |  |  |
| Mean age +/- SD | 61.4±12.9 | 65.8±13.9 | 0.109 |  |
| Sex ratio (men/women) | 2.5 (15/6) | 2.2 (52/24) | 1 |  |
| Smoking history (%) | 10 (50) | 25 (39.7) | 0.579 |  |
| Diabetes mellitus (%) | 5 (27.8) | 25 (33.3) | 0.863 |  |
| Cirrhosis (%) | 0 (0) | 9 (12) | 0.202 |  |
| Other immunodeficiency (%) | 4/18 (22.2) | 22/76 (28.9) | 0.771 |  |
| Mean time from first symptom to diagnosis +/- SD | 18.8±18.4 | 28.8±28.4 | 0.102 |  |
| Spinal pain at diagnosis (%) | 20 (95.2) | 65 (85.5) | 0.453 |  |
| Fever at diagnosis (%) | 13 (61.9) | 41 (56.2) | 0.827 |  |
| Neurological signs at diagnosis (%) | 20 (95.2) | 66 (86.8) | 0.447 |  |
| Radicular pain | 3 (14.3) | 17 (22.4) | 0.55 |  |
| Spinal cord compression | 18 (85.7) | 49 (64.5) | 0.11 |  |
| Other | 3 (15.8) | 24 (31.6) | 0.36 |  |
| AIS A or B | 9 (47.4) | 21 (34.4) | 0.456 |  |
| AIS C or D | 9 (47.4) | 40 (65.6) | 0.249 |  |
| Level involvement (%) |  |  |  |  |
| Cervical | 9 (42.9) | 24 (31.6) | 0.481 |  |
| Thoracic | 11 (52.4) | 44 (57.9) | 0.839 |  |
| Lumbosacral | 6 (28.6) | 27 (35.5) | 0.943 |  |
| Multiple spinal involvement | 5 (23.8) | 25 (32.9) | 0.596 |  |
| Radiological findings (%) |  |  |  |  |
| epidural inflammation | 20 (95.2) | 76 (100) | 0.216 |  |
| epidural abscess | 9 (47.4) | 39 (54.2) | 0.787 |  |
| Mean CRP at diagnosis +/- SD | 200.4±101.9 | 207±119.2 | 0.964 |  |
| Micro-organism identification |  |  |  |  |
| Positive blood cultures | 17 (81) | 60 (78.9) | 1 |  |
| *Staphylococcus aureus* | 13 (61.9) | 49 (64.5) | 0.71 |  |
| Coagulase negative staphylococci | 1 (4.8) | 2 (2.6) |  |  |
| Streptococci | 4 (19) | 11 (14.5) |  |  |
| *Enterobacteriae* | 2 (9.5) | 10 (13.2 ) |  |  |
| Other | 1 (4.8) | 1 (1.3) |  |  |
| Polymicrobial | 0 (0) | 3 (3.9) |  |  |
| Surgery | 9 (45) | 54 (71.1) | 0.055 |  |
| Mean time before surgery+/- SD | 5.8±5.6 | 4.2±7.4 | 0.53 |  |
| Mortality at 3 months | 3 (14.3) | 9 (13) | 1 |  |

**Trial**, trial group: patient data were obtained from the previously published randomized trial NCT00764114 investigating optimal duration of treatment in PVO; **Retro**, retrospective group: patients with PVO and presenting with SND were included from the French Hospital Discharge Database (patient data collected between 2001 and 2013 from 8 medical care centres in France). Data are mean (+/- SD, standard deviation), or number (%). No significant between-group differences were noted in univariate analysis. Other immunodeficiency: immunosuppressive drugs, HIV infection, chemotherapy, or active neoplasm. NSAIDs: use of Non-Steroidal Anti-Inflammatory Drugs before diagnosis. Mean time before diagnosis: time between first symptoms and diagnosis of PVO. Fever was defined as a body temperature > 38° Celsius. The sum of the involved levels exceeded the total number of subjects because one patient could have multiple vertebral levels affected. AIS: ASIA impairment scale. Epidural inflammation was defined by contrast enhancement of epidural tissue on MRI, epidural abscess was defined as the presence of a collection of fluid-equivalent signal with ring enhancement on MRI. Mean time before surgery: time between motor weakness diagnosis and surgery.

**Supplement 2: Sensitivity analysis with multiple imputation: Factors associated with severe neurological deficit (SND) in patients with pyogenic vertebral osteomyelitis (PVO)**

|  | Final model | |
| --- | --- | --- |
|  | aOR [95% CI] | p |
| Multiple spinal involvement | 1.1 [1.03–1.3] | 0.01 |
| Lumbosacral level involvement | 1 | - |
| Thoracic level involvement | 1.3 [1.2–1.4] | <0.001 |
| Cervical level involvement | 1.2 [1.1–1.4] | <0.001 |
| *Staphylococcus aureus* | 1.1 [1.02–1.2] | 0.02 |
| Spinal epidural abscess | 1.5 [1.3–1.6] | <0.001 |
| CRP over 150 mg/L | 1.1 [1.04–1.2] | 0.006 |

Final model presented was developed by a selection of step-down approach using Akaike criterion with 297 controls and 97 cases with multiple imputation of missing data by chained equation using MICE package with the R-software. aOR: adjusted Odds Ratio, CI: Confidence interval
